# Education Research: More Than a Checkbox: Implementing a Narrative-Based Child Neurology Resident Assessment System

**DOI:** 10.1212/NE9.0000000000200330

**Published:** 2026-09-08

**Authors:** Christopher Latimer, Robert Thompson Stone, Elizabeth T. Troy

**Affiliations:** 1 Department of Pediatrics, Section of Neurology, University of Colorado School of Medicine, Aurora; and; 2 Department of Neurology, University of Rochester School of Medicine and Dentistry, NY.

## Abstract

**Background and Objectives:**

Frequent, high-quality feedback is essential for competency-based medical education, yet many graduate medical education assessment systems rely on rating scales and infrequent assessments, limiting actionable feedback. Narrative comments, recognized for their catalytic effect, remain underutilized. The objective of this study was to design and implement a narrative-based, competency-mapped assessment system within a child neurology residency program and evaluate the feasibility of this reform.

**Methods:**

The University of Colorado Child Neurology Residency Program redesigned its assessment system in 2023, guided by the Good Assessment framework and the Accreditation Council for Graduate Medical Education Child Neurology Milestones. The new system prioritized adaptability to clinical context, narrative specificity, and integration into existing MedHub workflows. Each form required selection of a competency to assess and justification through narrative comments. Faculty development sessions introduced the structure, expectations, and strategies for writing effective narrative comments. Primary outcomes included volume of assessments and narrative comments. Secondary measures included assessor diversity, time to completion, and distribution of competency selection. We conducted a pre-post analysis comparing a 6-month preintervention period (July–December 2023) with a 6-month postintervention period (July–December 2024).

**Results:**

In the first year of implementation, there were 13 residents. A total of 260 assessments were completed (mean 28.9 per resident), generating 816 narrative comments (mean 90.7 per resident). The median time to completion was 10.5 days (IQR 2–24). Forty-nine unique assessors contributed, including physicians (79.5%) and nonphysicians (20.5%). In the pre-post comparison, assessments increased by 129% (55 to 126), unique assessors by 106% (16 to 33), and narrative comments by 512% (49 to 300). Narrative comments addressed all mapped competencies, with Patient Care and Interpersonal Communication Skills most frequently represented.

**Discussion:**

A narrative-based, adaptable assessment system is feasible to implement at scale within a child neurology residency program and substantially increases the volume of workplace-based assessments. The system diversified assessors' perspectives and produced narrative comments across all competencies without compromising timeliness. Limitations include single-site design and absence of narrative quality assessment. Future work will focus on evaluating the quality of narrative comments and acceptability of this assessment methodology.

## Introduction

One of the earliest formal descriptions of feedback framed it as information about a learner's performance intended to guide improvement in subsequent encounters,^1^ underscoring its central role in formative assessment.^2^ Traditional approaches to assessment in medical education relied heavily on time-based metrics and knowledge-focused examinations, which primarily address the foundational layers of clinical competence—what a learner knows.^3^ Miller's pyramid provides this helpful structure for conceptualizing the progression moving from knowledge (knows), to application (knows how), to demonstration of skills in controlled settings (shows), and ultimately to performance in real clinical environments (does).^4^ The shift toward competency-based medical education (CBME) expanded expectations beyond knowledge acquisition (knows) to include direct observation of clinical work and assessment of a trainee's ability to do the work of practice, requiring integration of multiple competencies by faculty to assess the learner. In this contemporary model, workplace-based assessments (WBAs) play a central role by generating frequent, behavior-based observations that support learner growth^2,3^ and contribute to a broader programmatic assessment system in which multiple data points are synthesized over time to inform summative decisions.^5^ Incorporating perspectives from multiple observers further strengthens this system by enhancing the breadth and reliability of information used to assess trainee performance.^2,3,6,7^

Within this context, the Accreditation Council for Graduate Medical Education (ACGME) Common Program Requirements^8^ mandate residency programs to, at minimum, assess learner performance at the conclusion of each rotation. Before our reform, the University of Colorado Child Neurology residency program met this requirement, using end-of-rotation forms with Milestone^9^-based ratings and a single comment field. However, this design functioned primarily as a summative tool. It placed significant cognitive load on faculty, yielded limited actionable feedback, and required high-stakes judgments across numerous competencies. For the Clinical Competency Committee (CCC), sparse and nonspecific data made it challenging to track longitudinal progress across the Milestones.^9^ By contrast, CBME emphasizes frequent, low-stakes formative assessments as foundational to professional growth,^3,5^ an expectation that the prior system did not meet.

To guide improvements, the Good Assessment framework articulated by Norcini and colleagues^10,11^ offers a coherent philosophy for designing high-quality assessments. Its 7 elements—validity, reproducibility, equivalence, feasibility, educational effect, catalytic effect, and acceptability—represent the variables that together determine the credibility and value of an assessment. These elements do not carry equal weight across all contexts, noting the variable importance depending on purpose of the assessments and priorities of different stakeholders. In programmatic assessment systems, van der Vleuten^5^ underscores that each individual tool should be deliberately designed to fulfill a specific purpose, providing 1 useful data point that gains meaning only when combined with other observations across assessors, contexts, and time. For workplace-based formative assessments, this means prioritizing elements that advance learning, including validity (alignment with intended purpose), educational effect (motivation for trainee learning), catalytic effect (promotion of self-reflection for learner advancement), feasibility (integration within workflows), and acceptability (ensuring assessor engagement). Thoughtfully overlapping assessments of key behaviors across different clinical contexts and assessors groups strengthens the interpretability of the resulting data. This philosophy positions assessment not as a stand-alone measurement but as an integrated educational practice designed to stimulate learning, support self-regulation, and inform program improvement. These principles guided the redesign of our system and align closely with the goals of CBME.

Graduate medical education accrediting organizations in the United States^12^ and worldwide,^13-15^ and more specifically the American Boad of Pediatrics and the Association of Pediatric Program Directors within pediatrics,^16^ have endorsed WBAs, citing the growing validity and reliability evidence for their role in CBME.^17,18^ Despite this, implementation challenges persist. Widely adopted tools, such as the mini-CEX form^19^ and the Direct Observation of Clinical Care,^20^ face limitations, including reliance on numeric rating scales, lack of specialty-specific detail, and poor integration into workflows. The American Academy of Neurology introduced a template aligning neurology-specific competencies with quantitative assessment.^21^ However, numerical scales carry risks of grade inflation, variability in interpretation, and the halo effect, raising concerns about reliability. Conversely, narrative comments have gained attention for their catalytic effects on trainee learning^5,22^ and their value in informing summative decisions,^23^ yet most existing tools provide minimal narrative data. Previous efforts, such as relocating a single comment box,^24^ offer incremental improvements but fall short of comprehensive form design. Other targeted tools, such as clinical skills evaluations^25^ and outpatient synthesis and management forms,^26^ similarly lack rich narrative data and fail to provide the depth and specificity needed to meet the needs of modern trainees.

In response to these gaps, the University of Colorado Child Neurology Residency program undertook a targeted redesign of its assessment system. Educational leadership developed and implemented a narrative-driven, competency-mapped, multisource approach, grounded in the Good Assessment framework and CBME principles. This study represents the first in a series examining the impact of this educational innovation. Here, we evaluate the feasibility of the redesigned system.

### Objectives

The objective of this study was to evaluate the feasibility of a narrative-driven, adaptable assessment system that prioritizes contextual competency mapping, observation-based feedback, and multisource perspectives to generate specific, actionable assessments. We evaluated feasibility by examining assessment volume, narrative comment frequency, assessor diversity, and completion metrics after implementation at a single training site.

## Methods

### Study Setting

The assessment reform was implemented within the University of Colorado Child Neurology Residency Program for PGY-4 and PGY-5 residents. Preintervention data were collected from July to December 2023, and postintervention data spanned from January to December 2024.

### Assessment System Design

To ensure design integrity, we incorporated principles from the Good Assessment framework.^10,11^ The Association of Pediatric Program Directors Primer further informed design decisions.^27^

Assessment content was anchored in ACGME Child Neurology Milestones.^9^ To ensure comprehensive support for resident’ clinical and professional development, the Milestones were grouped into 2 domains: clinical competencies (patient care [PC] and medical knowledge [MK]) and professional competencies (Systems-Based Practice, Practice-Based Learning and Improvement, Professionalism, and Interpersonal and Communication Skills). The original 26 subcompetencies were condensed into 24 statements, each accompanied by 3–4 behavioral descriptors to guide assessors in reflecting on their direct observations of resident performance. Table 1 presents 2 example competencies. See supplementary material for the full set of competency structure and mapping.

**Table 1:** Structure for Competency Assessment With Behavioral Descriptors

Competency: Patient care 1	
Patient care 1: Neurologic history: Describe the resident's ability to obtain and communicate an appropriate history.	Consider the following examples • Review the medical record for pertinent history • Elicit details on history needed for decision making • Presents a pertinent history • Documents appropriate level of detail
Competency: Medical knowledge 4	
Medical knowledge 4: Diagnostic investigation: Describe the resident's diagnostic approach for a patient.	Consider the following examples • Prioritizes investigation based on acuity • Tiers a broad workup based on testing of highest yield and cost-effectiveness • Counsels families on indications, risks, and potential outcomes of testing

Each assessment form was tailored to its respective clinical environment, aligning competencies with the context in which they were most likely to be observed. A total of 14 forms were developed, specifically 4 comprehensive, 7 focused, and 3 team member assessments. Comprehensive forms addressed inpatient and outpatient settings, while focused forms were designed for procedures and elective rotations. Team member forms represented a new addition to the portfolio, aimed at capturing perspectives from the broader health care team. Competencies were mapped to appropriate environments with intentional overlap across forms and assessors to ensure diversity of perspectives. Comprehensive and focused forms assessed both clinical and professional development, whereas team member forms concentrated on professional competencies. Most of the focused forms, including Brain Death, Lumbar Puncture, and EMG/NC (electromyography/nerve conduction), were delivered upon request by the resident to an assessor who had observed their skills in that particular domain or after scheduled sessions during their Outpatient Block, such as Lumbar Puncture or EMG Clinic.

Each form included up to 3 sections.

1. Selection of competency strengths and areas for improvement.
2. Narrative comments to contextualize competency selection.
3. A scale-based rating of overall resident performance during experience.

For competency selection, assessors chose mapped competencies from a dropdown menu, allowing customization of each form to the specific clinical encounter. For example, if a faculty member observed a new epilepsy diagnoses visit where the resident recommended a tiered workup of EEG, MRI brain, and genetic testing, while also providing detailed patient-centered counseling on seizure precautions and safety, the faculty could adapt the assessment to select applicable competencies, such as Diagnosis and Management in the Outpatient Setting, Diagnostic Investigation, and Patient and Family Education. Narrative comments were required to justify the selected competencies. These comments were triangulated with a scale-based rating, where faculty rated resident performance along an expectations continuum, categorized as below, meeting, or exceeding expectations. An example of a comprehensive form is available in the supplementary material.

To support feasibility, the system was designed in collaboration with the education coordinator and faculty leaders. The forms were hosted on MedHub (Minneapolis, MN), leveraging its existing integration within institutional workflows. Assessment frequency varied by setting, ranging from weekly for inpatient rotations to quarterly for nursing staff, and reminder emails were sent by an education coordinator for those forms that had been pending for 2 weeks. A faculty leader in each clinical environment reviewed the applicable form design; for example, the inpatient medical director vetted the inpatient-specific forms.

### System Implementation

Faculty engagement was central to successful implementation. During the launch week, a faculty development session was held to introduce the new system to the entire neurology section. Topics included the importance of narrative feedback, strategies for writing high-quality comments, and a walkthrough of the redesigned forms. Faculty were not chosen as assessors by any novel selection criteria but were sent assessments forms following clinics or inpatient weeks where they worked with the trainee. As such, any inpatient faculty who work on teams with residents or any outpatient faculty assigned to resident clinics were expected to be able to assess the residents using these forms. For inpatient faculty, they were asked to assess the inpatient resident at the end of the inpatient week they shared. For outpatient faculty, they were asked to assess a resident if they worked with them in clinic during the last week of the resident's outpatient block. For our nonfaculty assessors, they were primary neurology nurses at the outpatient clinic site where the resident most frequently sees patients, as well as EEG technologists from the main inpatient hospital. To reinforce adoption, the program director and education coordinator met weekly with inpatient attendings to gather feedback on resident performance, prompting discussion on the role of direction observation in assessment. These 30-minute meetings, held either in person or remotely, prompted inpatient attendings to discuss each learner on their teams, focusing on meaningful observations of clinical performance that highlighted areas of strength and opportunities for improvement. These debrief sessions, previously a longstanding practice, had been discontinued in recent years. With renewed emphasis on resident assessment, they were reinstated to elevate the importance of feedback and observation as essential elements of medical education.

### Program Evaluation

Data were collected from existing resident assessment forms housed within the residency management platform MedHub (Minneapolis, MN). Each resident was assigned a unique identifier, and assessment forms were reviewed for form type, resident assessed, assessor job title, competency selection for strengths and areas for improvement, and time to completion.

The primary outcomes were the number of completed forms and narrative comments per resident. Narrative comments were quantified by counting the number of text boxes completed on each form. Additional measures of feasibility included frequency of form utilization, completion rate, time to completion, and the number of unique assessors contributing feedback. Form design was evaluated by examining the frequency of competency identification and the distribution of strengths and areas for improvement across assessments.

### Statistical Analyses

The data from the first year after implementation of the reform (January 2024–December 2024) were evaluated initially to assess descriptive statistics after the intervention, including means, proportions, and interquartile ranges when appropriate. Then, data from 6 months preintervention (July–December 2023) were compared with a period of 6 months postintervention (July–December 2024), after a roll-out phase with minor adjustments to the forms. The study variables were described as numbers with proportions and sample means or medians, with interquartile ranges when applicable.

### Artificial Intelligence Disclosure Statement

This manuscript represents the original work of the authors. Artificial intelligence tools were not used in the design of the assessment system, and all described content was developed independently by the authors. Microsoft Copilot was used solely to enhance the clarity and flow of the written text. The authors have reviewed the final manuscript and affirm its readiness for publication.

### Standard Protocol Approvals, Registrations, and Participant Consents

This study was approved by the University of Colorado Institutional Review Board under Protocol 24-2090 as secondary research. Because the data were retrospective and deidentified, participant consent was not required.

### Data Availability

The assessment system design and deidentified data will be made available on request by a qualified educator.

## Results

### Descriptive Analysis

In the study period, the cohort included 13 unique child neurology residents. The preintervention period included 9 residents: 4 PGY-4 and 5 PGY-5. The postintervention period also included 9 residents in each 6 month period, comprised of 4 PGY-5 residents, 5 residents who graduated from PGY-4 to PGY-5 during the study time, and 4 additional PGY-4 residents in the latter 6 months. Across the 18-month study period, 5 residents were present for the entire duration.

In the first year of implementation, 260 assessments were completed with a mean of 28.9 per resident. Comprehensive forms accounted for 68.5% (178/260) of collected assessments, followed by focused (49/260, 18.8%) and then team members (33/260, 12.7%). Table 2 presents additional details by type of form. The median time to completion was 10.5 days with an interquartile range of 2–24 days. There were 49 unique assessors, with physicians (39/49, 79.6%) being the most common. The overall completion rate for sent forms during the year was 96% (357/370).

**Table 2:** Assessment Forms—Grouped by Form Type and Frequency of Use by Total Number (Percentage of Total)

Comprehensive forms	Frequency, (%)
Inpatient days	91 (35)
Inpatient nights	27 (10.4)
Outpatient—primary	34 (13.1)
Outpatient—satellite	26 (10)

Team member forms	Frequency
EEG technologist	6 (2.3)
Nursing	11 (4.2)
Advanced practice provider	16 (6.2)

Focused forms	Frequency, (%)
Electroencephalogram	13 (5)
Lumbar puncture	4 (1.5)
Electromyography	3 (1.2)
Brain death	7 (2.7)
Difficult conversation	3 (1.2)
Elective–clinical	7 (2.7)
Elective—research	12 (4.6)

Over the first calendar year, narrative comments totaled 816 for the residency program with a mean of 90.7 narratives per resident. For overall selection, PC (303/816, 37.1%) was the most selected clinical competency, and interpersonal and communication skills (215/816, 26.3%) were the most selected professional competency. Table 3 presents the distribution of competency selection for strengths and areas for improvement. Notably, all available subcompetencies on the comprehensive, elective, and team member forms were selected by at least 1 assessor. For the summative expectations item, faculty selected exceeding (86/168, 51.2%) and meeting (80/168, 47.6%) expectations with similar frequency and only occasionally selected below expectations 2/168 (1.2%).

**Table 3:** Distribution of Strengths and Areas for Improvement by Competency Domain Postintervention for the Residency Program, 1y Postintervention—Total, Percentage

Competency	Competency frequency by areas of strength, (%)	Competency frequency by areas for improvement, (%)
Patient Care (PC)	105 (43)	41 (26)
Medical Knowledge (MK)	23 (9)	34 (21)
Interpersonal and Communication Skills (ICS)	73 (30)	33 (21)
Systems-Based Practice (SBP)	28 (11)	28 (18)
Problem-Based Learning and Improvement (PBL)	9 (4)	18 (11)
Professionalism (PR)	7 (3)	4 (3)

### Comparison Analysis

The preintervention and postintervention cohorts each included 9 child neurology residents. In comparing 6 months preintervention with postintervention, as shown in Figure, the total quantity of assessments within the residency program increased by 129% (55–126) with a notable expansion of unique assessors by 106% (16–33) and increase in narrative comments by 512% (49–300). At the resident level, there was a substantial increase across all metrics: Assessments per resident increased from 6.1 to 14.2, assessor volume per resident grew from 5.5 to 11.2, and narratives per resident increased from 5.4 to 45.3. In 6 months postintervention, there were 13.9 narratives per resident on the PC competencies, 42% of total narratives. There were 3.6 narratives per resident on MK (11%), 8.1 narratives per resident on Interpersonal and Communication Skills (24%), 4.9 narratives per resident on Systems-Based Practice (15%), 1.9 narratives per resident on Problem-Based Learning (6%), and 1 narrative per resident on Professionalism (3%). Preintervention assessments were limited to physicians (16/16, 100%), whereas the postintervention physician pool was expanded (27/33, 82%) and nonphysicians account for 18% (6/33) of the assessors. The completion rate for sent forms improved from 86% (107/125) preintervention to 97.3% (177/182) postintervention.

**Figure:**
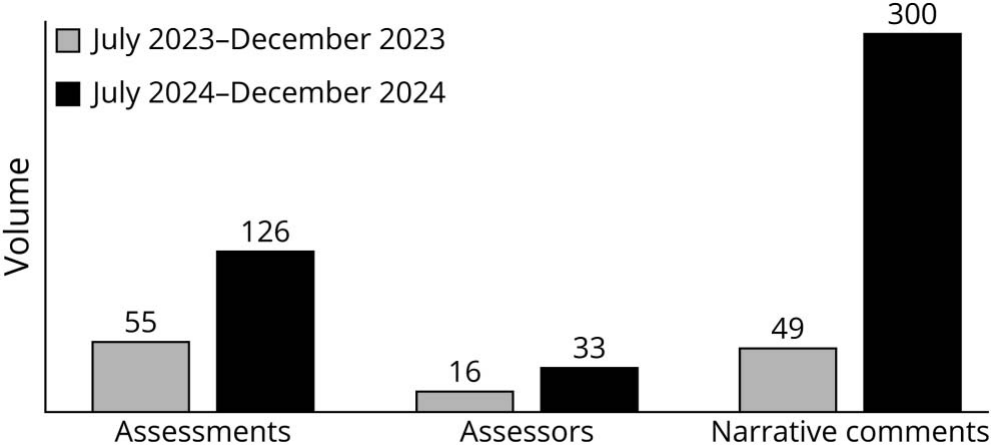
Volume of Assessments, Assessors, and Narrative Comments for the Residency Program, Preintervention and Postintervention

## Discussion

This study presents the first comprehensive assessment system described within a child neurology residency program. Our findings support that a narrative-based, adaptable assessment approach is feasible. The marked increase in assessment volume, narrative comments, and diversity of assessors demonstrates that a system emphasizing narrative feedback can be implemented at scale. These findings address key gaps in the prior system and align with the principles of the Good Assessment framework and CBME, highlighting the value of frequent, low-stakes observations that contribute to a richer programmatic assessment structure.

Workplace-based assessments have been widely adopted across health professions education and form the backbone of many competency-based training models. WBAs are typically operationalized through the Milestones and Entrustable Professional Activities (EPAs), which anchor assessments to clearly defined competencies and clinical tasks, respectively. Internationally, EPAs have been established for both adult^28,29^ and child^30^ neurology, providing structured expectations for direct observation and formative feedback. By contrast, in the United States, neither adult nor child neurology currently has ACGME-approved EPAs, despite prior calls for their development.^31,32^ Several adjacent fields, including pediatric critical care,^33^ developmental pediatrics,^34^ and adult neurology advance practice providers,^35^ have developed EPAs, offering examples of specialty-specific tasks. Because the field moves toward competency-based approaches, preparing training programs for eventual EPA adoption will require assessment systems capable of supporting observable, task-based judgements and generating high-quality narrative evidence. Until neurology-specific EPAs are fully defined, a Milestone-based system such as ours offers a practical structure for capturing meaningful workplace-based data and advancing a culture of guided performance development.

With this reform, assessments transitioned from comprehensive, high burden forms completed exclusively by physician faculty to adaptable, focused forms incorporating perspectives from the broader health care team. Although faculty had the option to select competencies that may have been easier to assess, assessors selected a wide range of competencies, suggesting alignment with actual clinical encounters. Notably, comparison analyses showed that residents received, on average, 1 to 14 comments per competency category, providing rich opportunities for CCC discussion. Areas for improvement were distributed evenly, except for professionalism, which was less frequently cited and may reflect gaps in assessor perspective. To optimize this domain, programs might expand the assessor pool to, for example, include education coordinators or consider alternative assessment modalities within the broader programmatic system. Because residents work within multidisciplinary teams, relying solely on physician faculty previously limited the scope of observed behaviors. Incorporating feedback from advanced practice providers, EEG technologists, and nurses offers a more holistic view of resident performance, particularly in communication, professionalism, and care coordination. Consistent with Kogan and Holmboe's recommendations,^3^ team member forms were restricted to competencies that these assessors were best positioned to evaluate. Existing literature suggests that reliability in workplace-based assessment improves with multiple observations, with stability often achieved with 4–8 assessments.^16,36^ Although some international programs specify minimum numbers of WBAs per competency or EPA, others allow greater flexibility given the variability to competence in CBME. Although our study did not aim to establish reliability thresholds, it illustrates where assessors naturally focus and underscores the value of accumulating observations across roles and settings. As the data set grows, patterns in competency selection will inform curricular needs and faculty development, while the adaptable form structure may help assessors refine observational skills.

Well-documented barriers to implementing frequent assessments have been described for both assessors and trainees.^37-41^ For physician faculty, challenges commonly include limited observation time,^42^ difficulty achieving faculty buy-in,^16,43^ and inadequate preparation for how to deliver high-quality feedback.^44^ Residents similarly report that the feedback is often insufficient or of poor quality, despite its recognized importance to professional development.^43,44^ Within neurology, medical student assessment literature identifies time as a common barrier, including time with the learner, time to complete forms, and time elapsed since the clinical encounter.^38^ Our program's uptake diverges from these reports, possibly reflecting the intentional design elements that prioritized feasibility. Focused, adaptable assessments reduced cognitive load; scheduled release in the learning management system supported administrative duties; and targeted faculty development helped standardize expectations for narrative structure. In this session, we focused on promoting a balanced approach of reinforcement of excellent behaviors and providing coaching on subtle changes to affect performance, ultimately in writing down content they had already discussed or debriefed in person. Weekly 30-minute program director debriefs with the inpatient teams helped to synthesize resident performance, prompting faculty to reflect on the week, having access to context from program director of skills they were working on, and then place this into comments for an assessment. This may further reinforce the perceived value of assessment, thereby increasing faculty engagement. These elements collectively help lower the stakes of individual assessments and distribute decision making across multiple observations. Although faculty satisfaction with the training session was not formally assessed, the notable uptake of the new system and increased assessment volume suggests that the training was effective.

Timeliness of feedback remains essential to effective WBA, yet the literature underscores that “timely” does not always mean immediate.^45^ Residents describe balancing the giver's emotional readiness, the learner's receptivity, and the context of the encounter when determining optimal timing. Both immediate verbal coaching and delayed written feedback can be meaningful when delivered with clarity and specificity. At the same time, delays beyond several days may introduce recall bias or reduce the educational impact. Our system aims to mitigate these challenges by supporting flexibility in form submission timing and thus prioritizing specificity over urgency in workflows. Although confidentiality considerations may prompt delayed or anonymized release in some programs, our design prioritizes transparency and learning value while maintaining respectful communication. By reinforcing expectations for specificity and supporting feasible completion processes, this system strives to balance timeliness, psychological safety, and accuracy. If performed well, the documentation of WBA during the course of a training has been aligned with a play—whereby residents grow and change through the lived experience which is residency.^46^

This study has several limitations. Because a pilot implemented at a single institution, the generalizability of the assessment design remains uncertain. However, components of the system have already been adapted by at least 3 other child neurology institutions, and future evaluations in diverse learning environments will help clarify its broader applicability. The system was developed within MedHub, which offers the technical flexibility to support this design, and it is unclear whether other learning management platforms possess similar capabilities. In addition, this study focused on quantitative metrics derived from the new assessment forms and does not fully capture the quality or acceptability of the feedback provided to residents. Finally, the weekly debrief sessions may represent a unique feature of our institution. Although these efforts are ongoing locally, it will be important to determine whether this assessment design can maintain high completion rates at other institutions without similar support.

The next phase of this work will expand beyond feasibility to examine both the catalytic effect and acceptability of this assessment design. An essential component will be to further evaluate the quality of narrative comments generated through this process. Although narrative comments are widely recognized as a valuable tool for enhancing assessment quality, their effectiveness depends on the specificity and relevance of the content.^16,26^ Faculty development is again essential to ensure that comments move beyond brief or vague statements and provide meaningful insights into resident performance. In undergraduate medical education, the Narrative Evaluation Quality Instrument (NEQI) has been developed and validated to assess the quality of narrative comments, including its use in child neurology clerkships.^47^ The NEQI evaluates comments based on performance domains, specificity, and learner usefulness. Faculty development sessions using this tool have demonstrated measurable improvements in narrative quality.^48^ As part of our ongoing work, we plan to adapt the NEQI for use in graduate medical education to further guide faculty in composing effective, learner-centered feedback, and in this subsequent work, we will present examples both helpful and unhelpful narrative comments to illustrate principles of this work.

In addition to evaluating narrative quality, a critical future direction will be assessing the acceptability of this methodology among faculty assessors. This includes exploring motivations for completing assessments, the influence of leadership support and weekly debriefs on faculty engagement, and the design features that most effectively promote participation. Because this framework has now been adopted at other institutions, a multicenter evaluation of acceptability will offer a more comprehensive understanding of the strengths, challenges, and transferability of this assessment methodology.

## Glossary

ACGME: Accreditation Council for Graduate Medical Education
CBME: competency-based medical education
CCC: Clinical Competency Committee
MK: Medical knowledge
NEQI: Narrative Evaluation Quality Instrument
PC: Patient Care
WBA: workplace-based assessment
